# Occupation and SARS-CoV-2 infection risk among 108 960 workers during the first pandemic wave in Germany

**DOI:** 10.5271/sjweh.4037

**Published:** 2022-08-31

**Authors:** Marvin Reuter, Mariann Rigó, Maren Formazin, Falk Liebers, Ute Latza, Stefanie Castell, Karl-Heinz Jöckel, Karin Halina Greiser, Karin B. Michels, Gérard Krause, Stefan Albrecht, Ilter Öztürk, Oliver Kuss, Klaus Berger, Benedikt MJ Lampl, Michael Leitzmann, Hajo Zeeb, Karla Romero Starke, Sabine Schipf, Claudia Meinke-Franze, Wolfgang Ahrens, Andreas Seidler, Bianca Klee, Tobias Pischon, Andreas Deckert, Börge Schmidt, Rafael Mikolajczyk, André Karch, Barbara Bohn, Hermann Brenner, Bernd Holleczek, Nico Dragano

**Affiliations:** 1 Institute of Medical Sociology, Centre for Health and Society, Medical Faculty and University Hospital, University of Düsseldorf, Dusseldorf, Germany; 2 Federal Institute for Occupational Safety and Health (BAuA), Berlin, Germany; 3 Helmholtz Centre for Infection Research, Braunschweig, Germany; 4 Institute for Medical Informatics, Biometry and Epidemiology (IMIBE), University Hospital Essen, Germany; 5 German Cancer Research Centre (DKFZ) Heidelberg, Div. of Cancer Epidemiology, Heidelberg, Germany; 6 Institute for Prevention and Cancer Epidemiology, Faculty of Medicine and Medical Center, University of Freiburg, Freiburg, Germany; 7 Institute for Biometrics and Epidemiology, German Diabetes Center, Leibniz Center for Diabetes Research at Heinrich-Heine-University ­Düsseldorf, Düsseldorf, Germany; 8 Institute of Epidemiology and Social Medicine, University of Münster, Münster, Germany; 9 Regensburg Department of Public Health, Germany; 10 Department of Epidemiology and Preventive Medicine, University of Regensburg, Germany; 11 Leibniz Institute for Prevention Research and Epidemiology - BIPS, Bremen, Germany; 12 Institute and Policlinic for Occupational and Social Medicine, Faculty of Medicine, Technische Universität Dresden, Germany; 13 Institute for Community Medicine, University Medicine Greifswald, Greifswald, Germany; 14 Institute for Medical Epidemiology, Biometrics and Informatics, Interdisciplinary Center for Health Sciences, Medical Faculty of the Martin Luther University Halle-Wittenberg, Halle (Saale), Germany; 15 Max Delbrück Center for Molecular Medicine in the Helmholtz Association (MDC), Molecular Epidemiology Research Group, Germany; 16 Heidelberg Institute of Global Health, Heidelberg University,Heidelberg, Germany; 17 NAKO e.V. Heidelberg, Germany; 18 Division of Clinical Epidemiology and Aging Research, German Cancer Research Center (DKFZ), Heidelberg, Germany; 19 Krebsregister Saarland, Saarbrücken, Germany; 20 Institute for Infectious Disease Epidemiology, TWINCORE, Hannover, Germany; 21 German Center for Infection Research (DZIF), Braunschweig, Germany; 22 Robert Koch Institute, Department for Epidemiology and Health Monitoring, Germany

**Keywords:** COVID-19, cohort study, infection risk at work, ISCO-08, KldB 2010, workplace

## Abstract

**Objective:**

The aim of this study was to identify the occupational risk for a SARS-CoV-2 infection in a nationwide sample of German workers during the first wave of the COVID-19 pandemic (1 February–31 August 2020).

**Methods:**

We used the data of 108 960 workers who participated in a COVID follow-up survey of the German National Cohort (NAKO). Occupational characteristics were derived from the German Classification of Occupations 2010 (*Klassifikation der Berufe 2010*). PCR-confirmed SARS-CoV-2 infections were assessed from self-reports. Incidence rates (IR) and incidence rate ratios (IRR) were estimated using robust Poisson regression, adjusted for person-time at risk, age, sex, migration background, study center, working hours, and employment relationship.

**Results:**

The IR was 3.7 infections per 1000 workers [95% confidence interval (CI) 3.3–4.1]. IR differed by occupational sector, with the highest rates observed in personal (IR 4.8, 95% CI 4.0–5.6) and business administration (IR 3.4, 95% CI 2.8–3.9) services and the lowest rates in occupations related to the production of goods (IR 2.0, 95% CI 1.5–2.6). Infections were more frequent among essential workers compared with workers in non-essential occupations (IRR 1.95, 95% CI 1.59–2.40) and among highly skilled compared with skilled professions (IRR 1.36, 95% CI 1.07–1.72).

**Conclusions:**

The results emphasize higher infection risks in essential occupations and personal-related services, especially in the healthcare sector. Additionally, we found evidence that infections were more common in higher occupational status positions at the beginning of the pandemic.

By 2022, the coronavirus disease (COVID-19) pandemic had led to over 320 million infections and 5.5 million deaths worldwide (1). Workplaces are generally considered to constitute a high-risk setting for virus transmission due to interpersonal contacts with clients, patients or colleagues who may be infected with SARS-CoV-2 (2, 3). Consequently, the analysis of occupation-specific infection risks is necessary to develop and tailor measures that aim to protect workers and reduce virus transmission in populations (4). However, due to rarity of occupational data among those tested for infection, investigations into occupational variation in infection risks are still scarce.

Generally, workers in essential occupations are supposed to be at highest risk for a SARS-CoV-2 infection (5). Essential workers ensure the supply of the population with elementary goods and services, thus facing greater infection risks due to physical contact or inability to work from home. Essential occupations include, for example, healthcare, logistics, transportation, police, jurisdiction, finance and insurance, or public administration (6). Yet, most studies available solely focus on healthcare workers and report above-average incidences of infection in this group compared with the general population (7–9). Considerably less is known about infection risks in other occupations. An analysis based on the UK Biobank indicates higher rates of hospitalizations and COVID-19-related deaths in healthcare professions, social care, and public transportation during the first wave (10). Norwegian registry data reveals that positive SARS-CoV-2 tests were more likely among workers in healthcare and public transportation during the first wave, but also in gastronomy and teaching during the second wave (11). In The Netherlands, higher infection risks were observed in the hospitality sector, in public transportation and among hairdressers during the second wave (12).

Overall, occupational infection risks have been studied only in few countries yet. However, the interplay of contextual aspects of the country (including socio-structural characteristics, functioning of the health system) and the applied policy measures (eg, prevention measures, testing policies) is likely to cause country-specific differences. Therefore, further studies in different countries are necessary. We aimed to address this research gap for Germany, where currently available empirical evidence is limited to sick leave data of a small number of insurance funds (13).

In addition to previous studies, we analyze occupational differences with both a focus on major groups (horizontal differences) but also according to occupational status positions (vertical differences). The horizontal dimension classifies workers according to performed tasks and duties and creates occupational groups that have high in-group similarity (eg, jobs in healthcare, jobs in production, or jobs in sales) (14). Horizontal differences in infection risks may then result from varying degrees of proximity to others or different requirements for physical presence (2, 15). In contrast, a vertical dimension of occupation expresses different positions in social status hierarchy and can be measured by the formal vocational qualification (skill level) or by the degree of personnel responsibility (supervisory or managerial position) (16). In analogy to the well-established social gradient in health (17, 18) and the assumption that people in a lower status position are more often exposed to occupational hazards, it can be assumed that low-skilled workers or those without a leadership function might more often be exposed to SARS-CoV-2. However, an alternative assumption is also reasonable as individuals in higher occupational status positions are more likely to travel and have higher physical mobility, accompanied by more frequent personal contacts.

Taken together, in this study, we aimed to add knowledge on occupational differences in SARS-CoV-2 infection risks by providing first analyses for Germany based on a nationwide sample of 108 960 workers. We present a comprehensive analysis of occupational infection risks and study different horizontal and vertical occupational characteristics.

## Methods

We used data from the German National Cohort (NAKO), which is the largest population-based cohort study in Germany and has therefore high potential for investigating health-related consequences of the pandemic (19). Between 2014 and 2019, 204 895 men and women aged 20–69 years took part in the baseline examination. In 18 study regions, at least 10 000 people were randomly selected from the registers of the residents’ registration office, invited, interviewed and examined (20). The mean response for the baseline assessment was 18% (21). The study design foresees periodic follow-up surveys approximately every five years. The general focus of NAKO is on the causes of cardiovascular diseases, diabetes, cancer, neurological and mental illnesses as well as respiratory and infectious diseases. In addition, a wide range of socio-demographic and employment-related factors is measured (22).

Between 30 April–12 May 2020, all 197 834 participants who gave their consent to be contacted again were invited by letter or e-mail to participate in a COVID-19 survey that thematically focused on health-related consequences of the pandemic. From 30 April–20 November, 161 892 people completed the questionnaire (response 81.8%). For the purpose of this study, we linked data of this COVID-19 questionnaire with information about participants’ occupation obtained previously during the baseline interview. We hereby assumed that most of the participants’ occupation did not change between the baseline and COVID-19 questionnaire.

### Study sample

The sample used for the following analyses was restricted to participants who completed the COVID-19 questionnaire and were currently employed or self-employed. Classification of persons in employment was based on labor force status assessed with the COVID-19 questionnaire and based on the concept of the International Labor Organization (23). As we were interested in occupation-specific infection risks, participants in unemployment or being inactive (housemen, housewives, retirees, pupils, students) were excluded from the analyses. However, those unemployed only after the SARS-CoV-2 test or those not tested and unemployed for less than two months when completing the COVID-19 questionnaire were retained for the analysis. Furthermore, as our analysis focuses on the first pandemic wave in Germany, we excluded all subjects who reported a positive SARS-CoV-2 test before February (implausible) or after August. Finally, 108 960 individuals were included in the study sample. Information on how applying the inclusion and exclusion criteria affected the sample size can be found in the supplementary material www.sjweh.fi/article/4037, e-table 1.

**Table 1:** Sample description, stratified by SARS-CoV-2 test status. N=108 960 workers. [SD=standard deviation].

	Negative or not tested (N=108 556)				Positive test (N=404)				P-value ^a^
	
	N	%	Mean	SD	N	%	Mean	SD	
Person-time at risk (days)			109.6	16.5			61.8	19.6	<0.001
Age (years)			48.3	10.8			48.9	10.2	0.284
19–24	1366	1.3			2	0.5			0.516
25–29	6593	6.1			20	5.0			
30–34	8123	7.5			27	6.7			
35–39	7102	6.5			22	5.5			
40–44	10 848	10.0			46	11.4			
45–49	18 426	17.0			79	19.6			
50–54	21 962	20.2			75	18.6			
55–59	18 540	17.1			80	19.8			
60–64	11 401	10.5			38	9.4			
65–69	3120	2.9			12	3.0			
70–76	1075	1.0			3	0.7			
Sex									
Male	52 918	48.7			203	50.2			0.547
Female	55 638	51.3			201	49.8			
Migration background									
No	76 974	70.9			276	68.3			0.253
Yes	31 582	29.1			128	31.7			
Employment relationship									
Employee	93 951	86.6			336	83.2			0.047
Self-employed	14 605	13.5			68	16.8			
Working hours									
Full-time (≥35 hours)	75 407	69.5			275	68.1			0.544
Part-time (<35 hours)	33 149	30.5			129	31.9			

a Test for statistical significance of group differences by a two-sided t-test for continuous variables or by a Pearson chi-2 test for categorical variables.

### Variables

*Infection with SARS-CoV-2*. Infection with SARS-CoV-2 was assessed by two questions. The first question was: “Have you been tested for the corona virus once or several times in a doctor’s practice, in a test center or in a hospital since 1 February 2020?” Notably, performed tests were PCR tests as no antigen tests were available during this time frame. If participants replied with “yes”, a second question was posed, asking “Was at least one of the test results positive?” An infection with SARS-CoV-2 was defined as responding with “yes” to both questions. For those with a positive or negative test, a further question was posed asking for the date of the first test (if tested positive) or the date of the last test (if ever tested negative). This information was used to calculate the person-time at risk (see next paragraph). As different SARS-CoV-2 test rates may influence the likelihood for detecting infections, we report the number of conducted tests for each indicator of occupational grouping in supplementary e-figure 1.

**Figure 1:**
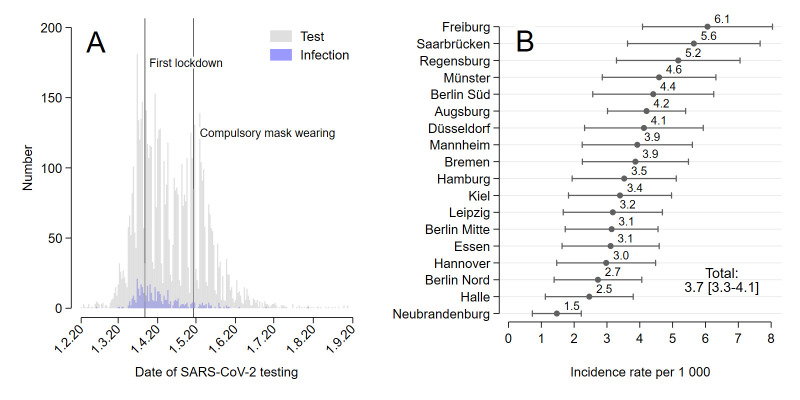
SARS-CoV-2 infections by (A) absolute numbers over time and (B) by incidence rates over study centre. Note: the first nationwide lockdown came into force on 22 March and included a ban on gatherings of more than two people not living in the same household. Futher measures included travel restrictions and the closure of schools, daycare centres and non-essential businesses (eg, pubs and cultural institutions). N=108 960 persons.

*Person-time at risk*. Since individuals were observed for different lengths of time (some completed the questionnaire earlier than others), the individual person-time at risk was calculated and taken into account in the multivariable analyses. The person-time at risk was the number of days between the 1 February 2020 and the date the COVID-19 questionnaire was filled in (for those not tested or tested negative) or the date of the SARS-CoV-2 test (in case a person was tested positive).

*Occupation*. Participants’ job title at the time of the baseline examination was ascertained by an open-ended question. A semi-manual coding procedure was applied by trained staff to convert responses into the five-digit occupational coding scheme German Classification of Occupations (*Klassifikation der Berufe* or KldB 2010) developed by the German Federal Employment Agency in cooperation with the German Federal Statistical Office (16). In addition, 5% of the answers were coded a second time in order to determine the reliability of the coding. Cohen’s Kappa yielded high reliability (weighted Kappa=0.90). The KldB 2010 was the basis for grouping individuals according to certain occupational characteristics (see below). For the purpose of international comparability, we additionally present the main analysis for major groups of the International Standard Classification of Occupations (ISCO-08) (14) in supplementary e-table 4.

*Occupational sectors and segments*. The horizontal dimension of occupational grouping was based on the first two digits of the KldB 2010 indicating the occupational main group. The KldB 2010 differentiates between 37 main groups that were qualitatively compiled according to specific job contents (eg, medical and healthcare occupations, occupations in production and processing of raw materials). As the large number of 37 main groups is not practicable for analytical purposes, we aggregated them in five occupational sectors and 14 occupational segments (24).

*Essential workers*. We also classified occupations according to whether they were considered essential (eg, doctors, pharmacists, transport workers, cashiers) or non-essential. Essential workers, ie, workers in essential occupations in times of the pandemic, were defined based on the first three digits of the KldB-2010 in accordance with previous studies that relied on the List of the Berlin Senate Administration from 17 March 2020 (25). Essential occupations with a low case number (<500) did not allow a precise estimation of incidences and were summarized into “others” (railway, aircraft and ship operation, health and safety administration, public health authority, traffic surveillance and control, building services and waste disposal). Essential workers were generally considered as jobs that ensure the supply of elementary goods and services in the population and therefore have an increased requirement for physical presence. In Germany, this includes finance and insurance as a sector that ensures the functioning of critical services (eg, cash supply and payment transaction, lending, processing of securities transactions) (6).

*Occupational skill level and supervisory/managerial responsibility*. We grouped occupations according to a vertical dimension by the required skill level and the degree of personnel responsibility. Occupational skill level was a variable with four categories derived from the fifth digit of the KldB 2010 differentiating between (i) unskilled or semiskilled occupations not requiring any formal training, (ii) skilled activities requiring vocational training, (iii) complex activities requiring further vocational training or a bachelor’s degree, (iv) and highly complex activities requiring an advanced tertiary degree. Supervisory and managerial responsibility was determined by the fourth digit of the KldB 2010 in combination with the denoted skill level. We differentiated between workers with (i) no supervisory or managerial tasks, (ii) supervisors and (iii) managers. Compared to supervisors, managers additionally have personnel and budget planning functions (eg, managing director, head of department).

*Control variables*. To control for possible confounding and to compare occupations with different socio-structural compositions, we controlled for age, sex, migration background, study center, weekly working hours, and the employment relationship (employee or self-employed) in multivariable analyses. We defined migration background if the interviewee reported that he or she or at least one of his or her parents was not born in Germany. To avoid over-adjustment bias, we have refrained from including health variables as smoking status or overweight, which are not considered as confounders but rather mediators in the relationship between occupation and infection (26).

### Statistical analysis

Several analytical steps were performed to investigate the relationship between occupation and SARS-CoV-2 infection risk. First, we described the incidence of positive test results in the cohort by plotting it against test date and study center.

Second, we described the sample in terms of socio-demographic and employment-related characteristics (stratified by SARS-CoV-2 test status). We used Pearson’s chi-squared test for categorical variables and two-sided t-test for continuous variables to investigate whether socio-demographic and employment-related characteristics significantly varied by test status (eg, whether self-employed were over- or underrepresented among the infected).

Third, we used modified Poisson regression analysis with robust variance estimation to compare incidence rates (IR) of SARS-CoV-2 infections by occupation (27). To handle that individuals were observed for different lengths in time, we specified person-time at risk as an exposure variable in each model. For each indicator of occupational grouping, we ran two regression analyses to calculate crude (unadjusted) IR as well as adjusted IR to control for possible confounding factors. Confounding factors were socio-demographic variables (age, sex, migration background, study center) and employment-related characteristics (weekly working hours and employment relationship). In case of horizontal indicators (occupational segment, occupational sector, and essential occupations), we additionally adjusted for skill level and supervisory/managerial responsibility in a subsequent model (to control that occupational sectors and segments might differ in their social composition, eg, higher share of low status jobs in cleaning or security). In case of vertical indicators (skill level and supervisory/managerial responsibility) were adjusted for occupational segment in a second model (to control that high status jobs might cluster within certain occupations that have higher incidences, eg, doctors in healthcare). Age was taken into account as a categorical variable (in five-year increments) in order to consider non-linearity in the relationship between age and infection. As a test of significance for multi-categorical variables, we calculated Wald tests.

Results of regression analyses were presented as IR for horizontal indicators and as incidence rate ratios (IRR) for vertical indicators. IRs for horizontal indicators were used as they do not require the definition of a reference category, which is somewhat arbitrary in a nominal categorical variable as occupation. Therefore, we converted regression estimates by a post-estimation command into adjusted predictions at the means (APM) along with respective 95% confidence intervals (CI), indicating the IR of SARS-CoV-2 infections per 1000 workers for average values of covariates (28). Recalculation to 1000 workers allows for a better presentation of small incidences (<1%).

Missing values in variables of interest (0.2–6.4%) were imputed by a predictive mean matching procedure. Tables showing the pattern of missing information (e-table 2) as well as a comparison of the original versus imputed data (e-table 3) can be found in the supplementary material. All analyses were performed using Stata 16.1 MP (64-bit, StataCorp LLC, College Station, TX, USA).

## Results

### Sample description

Among the 108 960 workers interviewed February–August 2020, 6062 (5.6%) reported to have been tested for SARS-CoV-2 at least once. Among them, 404 persons had a positive test result, resulting in a cumulative incidence in the sample of 0.37%. The mean person-time at risk was 109.5 days.

The mean age of participants was 48.3 years [standard deviation (SD) 10.8], 51.2% were women, and 29.1% had a migration background. As shown in table 1, those with a positive test did not differ from those untested or with a negative test in terms of age, sex, migration background, and working hours. However, self-employed participants were overrepresented among those tested positive.

Figure 1 gives a graphical visualization of (A) the number of tests and infections by test date and (B) the incidence rates by study center. The temporal distribution of infections follows the pattern in the general population during the first wave of the pandemic in Germany. Figure 1 also shows that the incidence tended to be high in the regions of the study centers Freiburg, Saarbrücken, Regensburg, Münster, and Berlin Süd and particularly low in Neubrandenburg.

### Multivariable analyses

Table 2 depicts IR of infections in the occupational sectors and segments. Accordingly, estimates were highest in jobs with personal services and business administration services and lowest in professions related to the production of goods. When taking into account the more detailed grouping of occupational segments, the highest IR were found in medical and non-medical healthcare occupations, cleaning services, agriculture, forestry and horticulture, and safety and security occupations. Generally, IR were below-average in the production sector and associated segments.

**Table 2:** Incidence rates (IR) of SARS-CoV-2 infections per 1000 workers (1 February–31 August 2020) by occupational sector and segment. N=108 960 employed individuals. [CI=confidence interval].

	Number (N)		Person-days ^a^	Unadjusted		Model 1 ^b^		Model 2 ^c^	
				
	Total	Cases	Mean	IR ^d^	95% CI	IR ^d^	95% CI	IR ^d^	95% CI
Occupational sector (1^st^ and 2^nd^ digit KldB 2010) ^e^									
Personal services	32 373	161	108.9	5.0	4.2–5.8	4.8	4.0–5.6	4.7	3.9–5.5
Business administration and related services	41 339	147	109.0	3.6	3.0–4.1	3.4	2.8–3.9	3.3	2.8–3.9
Other commercial services	7877	26	111.1	3.3	2.0–4.5	3.1	1.9–4.3	3.2	1.9–4.5
Service in the IT-sector and the natural sciences	6160	20	108.6	3.3	1.8–4.7	2.8	1.5–4.1	2.7	1.5–4.0
Production of goods	21 211	50	110.9	2.3	1.7–3.0	2.0	1.4–2.6	2.1	1.5–2.7
Occupational segment (1^st^ and 2^nd^ digit KldB 2010) ^e^									
Medical and non-medical healthcare	13 864	108	108.9	7.8	6.4–9.3	7.7	6.2–9.3	7.7	6.1–9.3
Agriculture, forestry and horticulture	1462	6	112.3	4.0	0.8–7.2	3.7	0.7–6.7	4.1	0.8–7.3
Safety and security occupations	2432	10	109.9	4.1	1.6–6.6	3.8	1.4–6.2	4.1	1.5–6.6
Cleaning services	753	3	113.7	3.8	-0.5–8.2	3.9	-0.5–8.3	3.8	-0.5–8.2
Business management and organisation	16 415	61	108.6	3.7	2.8–4.7	3.5	2.6–4.4	3.5	2.6–4.4
Business related service occupations	16 369	59	108.7	3.6	2.7–4.6	3.4	2.6–4.3	3.4	2.5–4.3
Commerce and trade	8431	27	110.2	3.2	2.0–4.4	3.0	1.8–4.1	3.0	1.8–4.1
Occupations in traffic and logistics	4759	14	111.2	2.9	1.4–4.4	2.6	1.2–3.9	2.8	1.3–4.3
Service in social sector and cultural work	15 291	46	108.4	3.0	2.2–3.9	2.9	2.1–3.7	2.6	1.8–3.5
Service in the IT-sector and the natural sciences	6094	19	108.6	3.1	1.7–4.6	2.6	1.4–3.8	2.5	1.3–3.6
Building and interior construction	5617	15	111.7	2.6	1.3–3.9	2.3	1.1–3.5	2.4	1.1–3.6
Manufacturing	4228	9	111.3	2.1	0.7–3.5	1.8	0.6–3.0	2.0	0.7–3.3
Food industry, gastronomy and tourism	3356	7	111.5	2.0	0.5–3.6	2.0	0.5–3.4	2.0	0.5–3.4
Occupations concerned with production technology	9889	20	110.1	2.0	1.1–2.9	1.7	0.9–2.4	1.7	0.9–2.5

a Person-days at risk were specified as an exposure variable to control for different lengths in observation times.

b Estimates of Model 1 were adjusted for age group (in five-year increments), sex, migration background, study centre, weekly working hours, and self-employment.

c Estimates of Model 2 additionally adjusted for occupational skill level (5th digit of the KldB 2010) and supervisory/leadership role (4^th^ digit of the KldB 2010).

d IR based on robust Poisson regression analysis. Separate models were calculated for each indicator.

e Wald chi-2 test for significance= P<0.001.

Table 3 shows that IR were higher among essential workers than among workers in non-essential occupations. After taking adjustment variables in Model 1 into account, essential workers were around twice as likely to report a positive test result. Table 3 also shows that risk of infection was higher in occupations with highly complex activities (requiring at least a four-year tertiary education). This inverse social gradient was still present when controlling for socio-demographic and employment-related factors in Model 1, and even when controlling for occupational segment in Model 2. The results also indicate that managers were more likely to contract a SARS-CoV-2 infection than workers without staff responsibility. In contrast, supervisors were less likely to have an infection compared with regular workers. However, differences by supervisory/managerial responsibility only reached the threshold for statistical significance for managers in the fully adjusted Model 2.

**Table 3:** Incidence rate ratios (IRR) of SARS-CoV-2 infections (1 February–31 August 2020) by essential occupations, skill level and supervisory/ managerial responsibility. N=108 960 employed individuals. [CI=confidence interval].

	Number (N)		Person-days ^a^	Unadjusted		Model 1 ^b^		Model 2 ^c^	
				
	Total	Cases	Mean	IRR ^d^	95% CI	IRR ^d^	95% CI	IRR ^d^	95% CI
Essential occupations									
Non-essential workers	75 502	224	109.6	1.00		1.00		1.00	
Essential workers	33 458	180	109.0	1.82	1.50–2.22	1.95	1.59–2.40	1.96	1.59–2.41
Skill level (5^th^ digit KldB 2010)									
Unskilled or semi-skilled	3 232	12	111.9	1.14	0.64–2.06	1.11	0.61–2.01	1.21	0.67–2.20
Skilled activities	46 330	148	110.2	1.00		1.00		1.00	
Complex activities	24 943	86	109.1	1.09	0.84–1.42	1.06	0.81–1.38	1.15	0.87–1.52
Highly complex activities	34 455	158	108.5	1.46	1.17–1.82	1.36	1.07–1.72	1.39	1.08–1.80
Supervisory/managerial responsibility (4^th^ digit KldB 2010)									
No	100 273	368	109.4	1.00		1.00		1.00	
Supervisor	3 359	8	110.1	0.64	0.32–1.30	0.63	0.31–1.28	0.72	0.36–1.47
Manager	5 328	28	109.6	1.43	0.97–2.10	1.38	0.94–2.03	1.51	1.01–2.24

a Person-days at risk were specified as an exposure variable to control for different lengths in observation times.

b Estimates of Model 1 were adjusted for age group (in five-year increments), sex, migration background, study centre, weekly working hours, and self-employment.

c Estimates of Model 2 were additionally adjusted for skill-level and supervisory/managerial responsibility (for the variable essential occupations) or for occupational segment (1st-2nd digit of the KldB-2010) (for the variables skill level and supervisory/managerial responsibility).

d IRR based on robust Poisson regression analysis. Separate models were calculated for each indicator.

**Figure 2:**
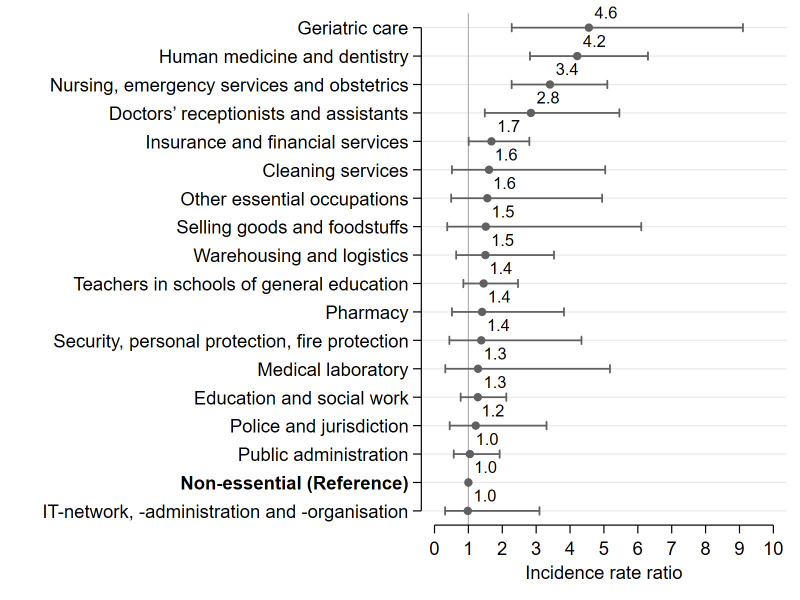
Risk for SARS-CoV-2 infection (1 February–31 August 2020) among different groups of essential workers in comparison to non-essential workers. Incidence rate ratios obtained from robust Poisson regression analysis (person-time at risk specified as an exposure variable to control for different observation times). Estimations were adjusted for age group (in five-year increments), sex, migration background, study centre, weekly working hours, self-employment, occupational skill level (5^th^ digit of the KldB-2010), and supervisory/leadership role (4^th^ digit of the KldB-2010). N=108 960 employed individuals.

Additionally, we plotted IRR using non-essential workers as the reference category (figure 2). When comparing essential to non-essential workers, we found that the risk of infection was more than four times as high among medical doctors, dentists, and geriatric nurses compared with non-essential workers. Further, employees working in nursing and ambulance or as doctoral assistants had around three-fold the risk of being infected compared to non-essential workers. The figure also reveals that workers in insurance and financial services were 1.7 times as likely of being infected as non-essential workers.

### Sensitivity analyses

We estimated infection rates by ISCO major groups as an alternative occupational coding scheme (see supplementary e-table 4). We found the highest IR among technicians and associate professionals (IR 4.9, 95% CI 4.0–5.8), followed by managers (IR 3.7, 95% CI 2.2–5.3), and the lowest rates in craftsmen and related trades workers (IR 1.6, 95% CI 0.7–2.6), as well as among machine operators and assemblers (IR 1.2, 95% CI -0.0–2.3). As different SARS-CoV-2 test rates may influence the likelihood for detecting infections, we show the frequency of infections and tests conducted in each occupational group (see supplementary e-figure 1). Test were more likely in medical occupations, but did not systematically differ between other occupational groups.

## Discussion

In this study, we compared SARS-CoV-2 infection rates between occupational sectors and segments and explored whether infection rates varied by skill level and leadership position. Based on a cohort including over 100 000 workers, this study is the first in Germany using survey data to complement previous analyses of health insurance registers (13) and studies based on ecological designs (29).

During the first pandemic wave, we found that infection rates differed by occupational sector, with the highest IR in personal services and business administration services and the lowest rates in occupations related to the production of goods. When taking into account the more detailed grouping of occupational segments, we found the highest IR in medical and non-medical healthcare, safety and security occupations, business management and organization, and business-related service occupations. Rates were also above-average among cleaning services, however, due to the comparatively low number of cases in this group, the estimate is less stable as indicated by the wide confidence interval. Medium-level infection rates were found in commerce and trade, traffic and logistic, service in the social sector and cultural work, and service in the IT-sector and natural science. We observed the lowest IR in building and interior construction, manufacturing, food industry, gastronomy and tourism, and occupations concerned with production technology.

As expected, healthcare and personal services were at highest risk for infection, which is in line with the considerations that proximity to others, especially infected people, is a main risk factor (2, 15). Elevated infection rates in personal services, especially in healthcare and geriatric care, were also observed in German health insurance data (13), in Norwegian register data (11), among participants of the UK Biobank study (10), in a prospective cohort of healthcare workers from the UK and the USA (7), and in a sample of Swiss workers (9). In addition, we also found comparatively high infection rates in business management and business-related services. Other studies did not report this finding as they only accounted for categories of essential occupations without looking at other major groups (11, 12). An exception is the UK Biobank study that investigated differences in infection risks between a wide range of major occupational groups during the first pandemic wave (10). In our sensitivity analyses we used a similar grouping, based on the ISCO-08 major groups, and a similar set of control variables. Here we observed notable differences when comparing Germany with the UK. In both studies, the highest rates were observed among professional and technical occupations. However, managers were found to be at high risk in Germany, in contrast to the UK where managers belonged to the group at lowest risk. In addition, a study from Switzerland observed an above-average prevalence of SARS-CoV-2 IgG antibodies in managers and assistant managers during the first wave (9). Higher infection rates among German and Swiss managers might be a result of recreational ski trips, which are mostly carried out by people of higher socio-economic position and have been discussed as one of the main drivers of virus transmission in Germany during the first wave (30).

As a second main result, we observed the lowest risk for infection in manufacturing and production-related occupations. Low rates in food production were also found with regard to sickness absence notes for Germany during the period of January–May 2020 (13). However, a contrary pattern was observed in the UK and Canada, were hospitalization and workplace outbreaks were more common in process, plant and machine operators (10), as well as in manufacturing, agriculture, forestry, fishing, and hunting (31). While we also observed high infection rates in agriculture, forestry and horticulture, we did not find evidence for elevated infections risks in manufacturing in the NAKO during the first wave in Germany. A possible explanation for country differences might be the introduction of short-time work regulation that also affected jobs in the production sectors in Germany.

The third main result of our study is the inverse social gradient between occupational position and the risk of a SARS-CoV-2 infection. We estimated a higher risk in occupations requiring an advanced tertiary degree and among persons occupying a managerial position. For Germany, these results corroborate the findings of ecological studies that found higher infection rates in high-income regions during the first wave (32). An explanation behind might be the higher mobility of persons in high-income jobs at the beginning of the pandemic when travel restrictions were not in place yet. Recreational ski trips mostly carried out by persons with higher socio-economic position were discussed as a relevant factor contributing to the spread of the virus in the first wave (30). However, it is also documented that in the second, and especially in the third wave, the relationship has reversed as high-status jobs were more likely the ones which were shifted into remote work (32).

### Limitations and strengths

During the first wave of the pandemic, lockdown measures led to shop closures and forced a part of the workforce into remote work (eg, this concerned teachers or service staff in restaurants and bars). Thus, reported infection rates in occupations that were affected by the lockdown do not truly reflect the risk of infection. A further point is that observed associations should not be generalized to subsequent phases of the pandemic, as several conditions have changed later on, including the occurrence of new virus variants, the implementation of workplace safety measures and the roll-out of vaccines. Another point is that it is unclear what share of infections in our population was transmitted during work, as we have no information where an infection originated. Furthermore, test rates varied by occupation and were considerably higher in medical occupations. Thus, we could underestimate infection risks in non-medical occupations. Moreover, our analysis is based on the occupation held at time of the baseline examination. Although studies indicate a low degree of occupational mobility over time (only around 4% of German workers change their occupation per year) (33), this might have biased downward the size of risk estimates to some degree. Although we considered separate analyses for males and females, the number of infections was too low (N=404) and stratification would result in insufficient statistical power. A last point is that NAKO baseline response was quite low (18%), which is most likely due to the comprehensive baseline examination and the general trend of declining survey response in developed countries (34). Although unit nonresponse can affect estimation of incidences, it has less impact on the association between occupation and infection, which was the main interest of this study (35, 36).

Apart from these limitations, this study has several strengths. For the first time it was possible to link individual data on SARS-CoV-2 infection risks with occupation-related information based on a population-based study in 18 study centers in 13 federal states of Germany. The large number of cases in the NAKO allowed a comparatively robust estimation even of a rare event such as SARS-CoV-2 infection during the first wave. The extensive processing of occupational information allowed a large number of different occupational characteristics to be examined. Furthermore, by taking into account many socio-demographic and occupational covariates, important confounders could be controlled for. As occupational differences in infection rates remained robust even after adjustment for a wide range of confounder, this speaks in favor of robustness of the main findings.

### Concluding remarks

This study yields important insights into occupational SARS-CoV-2 infection risks in Germany for the first pandemic wave. Our results reinforce previous empirical evidence emphasizing higher infection risks among workers in essential occupations and personal-related services, especially in the healthcare sector. Additionally, we found evidence that infections were more common in higher occupational class positions at the beginning of the pandemic.

### Funding

The Federal Institute for Occupational Safety and Health (BAuA; Germany) financially supported the scientific realization of this analysis (Grant BAuA-F2515). The analysis was conducted with data from the German National Cohort (GNC). The GNC is funded by the Federal Ministry of Education and Research (BMBF) [project funding reference numbers: 01ER1301A/B/C and 01ER1511D], the federal states and the Helmholtz Association with additional financial support by the participating universities and the institutes of the Helmholtz Association and of the Leibniz Association. We thank all participants who took part in the GNC study and the staff in this research program.

### Conflicts of interest

The authors declared no conflict of interest concerning the research, authorship, and publication of this article.

### Protection of research participants

Written informed consent was obtained from all participants included in the study. An external ethics advisory board has been established that accompanies NAKO over the full study period. A ‘Code of Ethics’ of NAKO (*Ethikkodex*) has been developed and the study is under steady surveillance by the ethical committees of the regional study centers (20).

### Availability of data and materials

The datasets analyzed during the current study are not publicly available due to privacy concerns but are available from the corresponding author on reasonable request.

## Supplementary material

Supplementary material
