# Supplementary material for: Occupation and SARS-CoV-2 infection risk among 108 960 workers during the first pandemic wave in Germany
Source: Scand J Work Environ Health. 2022 Aug 31;48(6):446–56. doi: 10.5271/sjweh.4037 (PMC9888438; doi:10.5271/sjweh.4037)
Supplement: Supplementary material [file SJWEH-48-446-S001.pdf]

Occupation and SARS-CoV-2 infection risk among 108 960 workers during the first pandemic wave in Germany[1]

by Marvin Reuter, DrPH,[2] Mariann Rigó, PhD, Maren Formazin, PhD, Falk Liebers, MD, Ute Latza, PhD, Stefanie Castell, MD, Karl-Heinz Jöckel, PhD, Karin Halina Greiser, MD, Karin B. Michels, PhD, Gérard Krause, MD, Stefan Albrecht, MA, Ilter Öztürk, MA, Oliver Kuss, PhD, Klaus Berger, MD, Benedikt MJ Lampl, MD, Michael Leitzmann, MD, Hajo Zeeb, MD, Karla Romero Starke, PhD, Sabine Schipf, PhD, Claudia Meinke-Franze, PhD, Wolfgang Ahrens, PhD, Andreas Seidler, MD, Bianca Klee, MPH, Tobias Pischon, MD, Andreas Deckert, PhD, Börge Schmidt, PhD, Rafael Mikolajczyk, MD, André Karch, MD, Barbara Bohn, PhD, Hermann Brenner, MD, Bernd Holleczek, MD, Nico Dragano, PhD

1. Supplementary material
2. Correspondence to: Dr. Marvin Reuter, Institute of Medical Sociology, Centre for Health and Society, Medical Faculty and University Hospital, Heinrich Heine University Duesseldorf Moorenstrasse 5, 40225 Düsseldorf, Germany. [E-Mail: marvin.reuter@uni-duesseldorf.de]

e-Table 1. Eligibility criteria for inclusion in the study sample

	Included		Excluded
	N	%	N
Baseline sample (2014-2019)	204 895	100.0	-
Invited for participation in the COVID follow-up (2020)	197 834	96.6	7 061
Completion of the questionnaire (2020)	161 849	79.0	35 985
Participants not unemployed or inactive at time of the COVID-19 questionnaire	109 328	53.1	52 521
If tested for SARS-CoV-2: Test date not before 01.02.2020 or after 31.08.2020	108 960	52.9	368
**Study sample**	**108 960**	**100.0**	

e-Table 2. Missing values in variables of interest

		Missing		Complete		Total	
**Variable**	**Label**	**N**	**%**	**N**	**%**	**N**	**%**
**Dependent variable**							
a_postest	Infection with SARS-CoV-2	272	0.2	108 611	99.8	108 883	100.0
**Independent variables**							
timerisk	Person-time at risk	0	0.0	108 960	100.0	108 960	100.0
age	Age in years	0	0.0	108 960	100.0	108 960	100.0
gender	Sex	0	0.0	108 960	100.0	108 960	100.0
a_migr	Migration background	34	0.0	108 926	100.0	108 960	100.0
studz	Region of study centre	0	0.0	108 960	100.0	108 960	100.0

a_workh	Weekly working hours	7 322	6.7	101 638	93.3	108 960	100.0
selfemp	Employment relationship	2 381	2.2	106 579	97.8	108 960	100.0
a_kldb_major	KldB 2010 major group	5 380	4.9	103 580	95.1	108 960	100.0
a_kldb_anf	KldB 2010 skill level	5 380	4.9	103 580	95.1	108 960	100.0
a_kldb_fuehr	KldB 2010 sup./manager responsibility	5 380	4.9	103 580	95.1	108 960	100.0
a_kldb_sek	KldB 2010 Occupational sector	5 380	4.9	103 580	95.1	108 960	100.0
a_kldb_seg	KldB 2010 Occupational segment	5 380	4.9	103 580	95.1	108 960	100.0
essential	Essential workers	5 380	4.9	103 580	95.1	108 960	100.0
a_isco_major	ISCO-08 major group	6 223	5.7	102 737	94.3	108 960	100.0
**Auxiliary variables**							
a_househ	Household composition	8	0.0	108 952	100.0	108 960	100.0
a_covtest	Test for SARS-CoV-2	272	0.2	108 688	99.8	108 960	100.0
a_homeoffice	Working from home	0	0.0	108 960	100.0	108 960	100.0
a_riskcont~t	Risk contact to an infected Person	0	0.0	108 960	100.0	108 960	100.0
a_red_whrs	Reduction in working hours	0	0.0	108 960	100.0	108 960	100.0
a_inc_whrs	Increase in working hours	0	0.0	108 960	100.0	108 960	100.0
a_newjob	Job change	0	0.0	108 960	100.0	108 960	100.0
a_shorttim~l	Receiving of short-time allowance	0	0.0	108 960	100.0	108 960	100.0
a_terminat~n	Dismissal	0	0.0	108 960	100.0	108 960	100.0
a_noworkch~s	No work changes	0	0.0	108 960	100.0	108 960	100.0

e-Table 3. Comparison of original versus imputed data

	Original					Imputed				
**Variable**	**N**	**Mean**	**SD**	**Min**	**Max**	**N**	**Mean**	**SD**	**Min**	**Max**
a_migr	108 926	0.29	0.45	0	1	108 960	0.29	0.45	0	1
a_househ	108 952	2.64	1.28	1	6	108 960	2.64	1.28	1	6
a_workh	101 638	1.30	0.46	1	2	108 960	1.31	0.46	1	2
selfemp	106 579	0.14	0.34	0	1	108 960	0.13	0.34	0	1
a_kldb_major	103 580	6.01	2.25	0	9	108 960	5.99	2.26	0	9

a_kldb_anf	103 580	2.83	0.91	1	4	108 960	2.83	0.91	1	4
a_kldb_fuehr	103 580	1.13	0.46	1	3	108 960	1.13	0.46	1	3
a_kldb_sek	103 580	2.52	1.08	1	5	108 960	2.51	1.09	1	5
a_kldb_seg	103 580	7.53	2.94	1	14	108 960	7.49	2.96	1	14
essential	103 580	5.20	9.84	1	99	108 960	5.17	9.83	1	99
a_isco_major	102 737	3.39	1.88	0	9	108 960	3.38	1.88	0	9
a_covtest	108 688	0.06	0.23	0	1	108 960	0.06	0.23	0	1
a_postest	108 688	0.00	0.06	0	1	108 960	0.00	0.06	0	1

e-Table 4. Incidence rates (IR) of SARS-CoV-2 infections per 1 000 workers (01.02-31.08.2020) by occupational major group (ISCO)

	Number (N)		Person-days (x̄)	Unadjusted		Model 1		Model 2	
	Total	Cases		IR	95% CI	IR	95% CI	IR	95% CI
**ISCO major groups (1st digit ISCO)**									
Technicians and associate professionals	27 292	127	109.0	4.7	3.9–5.5	4.5	3.7–5.3	4.8	3.9–5.7
Managers	7 752	39	109.4	5.0	3.5–6.6	4.3	2.9–5.7	3.7	2.2–5.3
Armed forces occupations	326	1	110.7	3.0	-2.9–9.0	3.3	-3.3–10.0	3.4	-3.3–10.1
Professionals	35 947	141	108.3	4.0	3.3–4.6	3.6	3.0–4.2	3.3	2.6–4.0
Skilled agricultural, forestry and fishery workers	886	3	112.4	3.3	-0.4–7.0	3.1	-0.4–6.5	3.2	-0.4–6.9
Clerical support workers	13 772	40	109.0	2.9	2.0–3.8	2.8	1.9–3.7	2.9	2.0–3.8
Services and sales workers	10 594	30	111.1	2.8	1.8–3.8	2.7	1.7–3.7	2.8	1.7–3.9
Elementary occupations	1 932	7	112.7	3.5	0.9–6.1	3.3	0.8–5.8	2.6	0.0–5.2
Craft and related trades workers	7 252	12	112.5	1.6	0.7–2.5	1.5	0.6–2.4	1.6	0.7–2.6
Plant and machine operators and assemblers	3 207	4	112.8	1.2	0.0–2.4	1.1	0.0–2.3	1.1	-0.0–2.3
Wald chi2 test for significance				p < 0.001		p = 0.001		p = 0.001	

IR=Incidence rate. x̄=Mean. CI=Confidence interval. IRs based on Poisson regression analysis. Person-days at risk were specified as an exposure variable to control for different lengths in observation times. Estimates of Model 1 were adjusted for age group (in five-year increments), sex, migration background, study centre, weekly working hours, and self-employment. Estimates of Model 2 were additionally adjusted for occupational skill level (5th digit of the KldB 2010), and supervisory/managerial responsibility (4th digit of the KldB 2010). N=108 960 employed individuals.

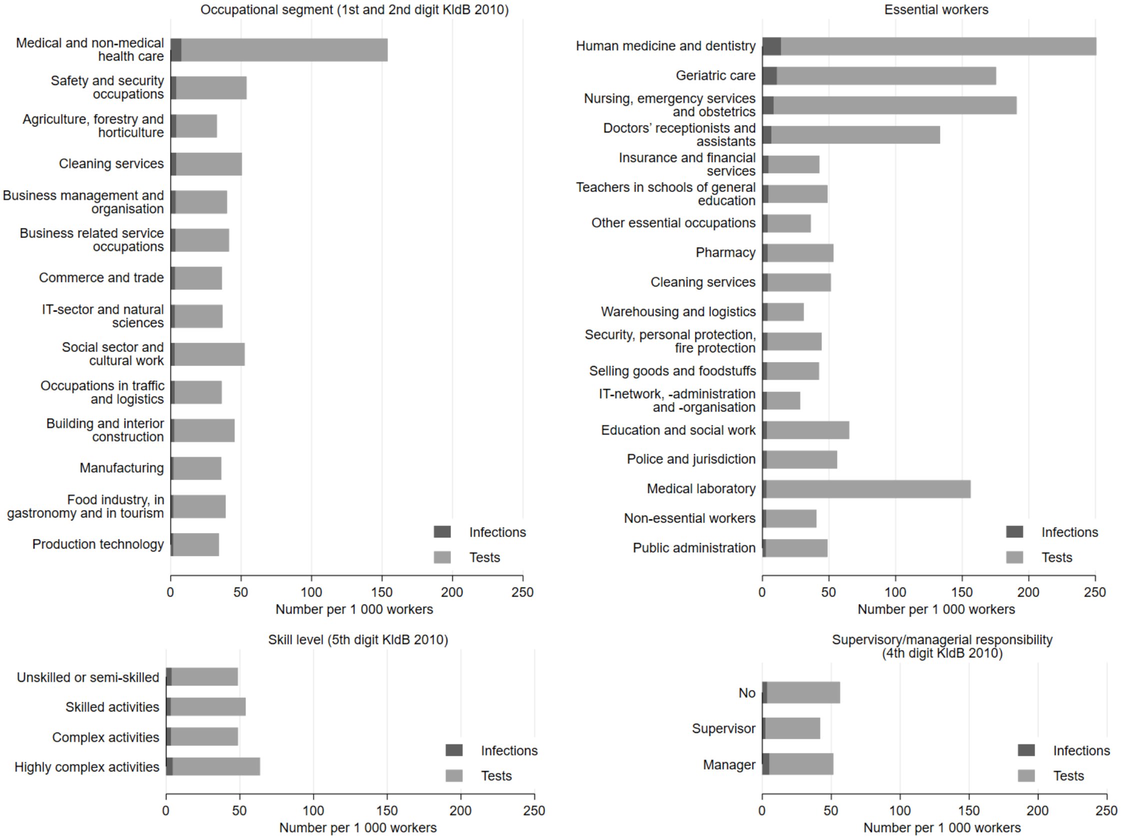

e-Figure 1. Cumulative number of SARS-CoV-2 tests and infections by occupational segment, essential occupation, skill level and supervisory/managerial responsibility.

N=108,960 employed individuals. Cumulative numbers per 1000 workers in each category of occupational grouping.
